# Immediate effect of two yoga-based relaxation techniques on attention in children

**DOI:** 10.4103/0973-6131.72632

**Published:** 2010

**Authors:** Balaram Pradhan, HR Nagendra

**Affiliations:** Division of Yoga and Life Sciences, Swami Vivekananda Yoga Anusandhana Samsthana, Bangalore - 560 019, India

**Keywords:** Cancellation, meditation, relaxation, yoga

## Abstract

**Aims:**

To investigate the effect of two yoga-based relaxation techniques, namely, cyclic meditation (CM) and supine rest (SR), using the six letter cancellation task (SLCT).

**Materials and Methods:**

The subjects consisted of 208 school students, (132 boys, 76 girls) in the age range of 13 – 16 years. The subjects were assessed on SLCT before and immediately after both yoga-based relaxation techniques.

**Results:**

After both practices, the total and net scores were significantly increased, although the magnitude of change was more after CM than after SR in the net scores (14.5 versus 11.31%). The net score change in the CM session was significantly larger than the change in the SR, whereas, there was no significant change in the wrong cancellation score. After either practice, the total and net scores were significantly increased, irrespective of gender and age.

**Conclusions:**

Both CM and SR led to improvement in performance, as assessed by SLCT, but the change caused by CM was larger than SR.

## INTRODUCTION

A yoga practice derived from an ancient Indian yoga text (*Mandukya Karika*) called cyclic meditation (CM) is a technique that combines ‘stimulating’ and ‘calming’ practices, based on a statement in an ancient yoga text suggesting that such a combination may be especially helpful to reach a state of mental equilibrium, which consists of the practice of physical postures interspersed with relaxation, which has been used for stress relief.[1] After this practice there was a significant reduction in oxygen consumption when compared to an equal period of supine rest in *shavasana*.[2,3]

Recent studies on CM have suggested that sympathetic activation occurs predominantly during the yoga posture phases of CM, whereas, following CM, the parasympathetic nervous system becomes dominant.[4] The results support the idea that a combination of yoga postures with supine rest (in CM) reduces the energy expenditure compared to supine rest alone and[5] CM enhances the cognitive processes underlying the generation of P300.[6] CM brings about a greater improvement in the performance of the cancellation task.[7] To avoid the effect of memory during repeated administration, parallel worksheets have been prepared, by changing the sequence of letters randomly in the working section, which was not taken care of in the earlier study.[8]

Hence, the present study aims to evaluate the SLCT performance on school students, following two yoga-based relaxation practices.

## MATERIALS AND METHODS

### Subjects

Two hundred and eight school students were selected in the present study with an age range between 13 and 16 years (*M*=13.84; *SD*=0.98). All of them were healthy and proficient in English. They were trained for practicing both CM and SR for seven days. The participants were excluded from the study if they had a history of neurological or psychiatric disturbances, were younger than age 12 or older than 16 years of age, under medication, had a history of learning disability, or were not proficient in English. After a complete description of the study, the participants had given their written informed consent.

### Procedure

The participants were assessed in two types of sessions, namely, CM and SR. For half the subjects, the CM session took place on one day with SR the next day. The other subjects had the order of the sessions reversed. The subjects were alternately allocated to either schedule, to prevent the order of sessions influencing the results. Each session had a duration of 22 minutes and 30 seconds. Assessments were made immediately before and after each session.

### Instrument

The six-letter cancellation task consisted of a test worksheet that specified the six target letters to be cancelled and had a ‘working section’ that consisted of letters of the alphabet arranged randomly in 14 rows and 22 columns. The participants were asked to cancel as many six target letters as possible, which were printed at the top of working section of the test sheets, in the specified time, that is, 1 minute 30 seconds. They were told that there were two possible strategies, that is, (i) doing all six letters at a time, or (ii) selecting any one target letter out of the six. They were asked to choose whichever strategy suited them. They were also told that they could follow a horizontal, vertical or a random path according to their choice.[8] The scoring was done by a person who was unaware when the assessment was made, whether the participant was engaging in CM or SR, and whether the assessment was ‘pre’ or ‘post’ the session. The total number of cancellations and wrong cancellations were scored and the net scores were calculated by deducting the wrong cancellations from the total cancellations attempted. As this test was administered before and immediately after the intervention, parallel work sheets were prepared by changing the sequence of the letters randomly in the working section. Hence, the subjects were divided in two sessions in equal numbers and altered the next day. Both the sessions received one set of worksheets before a session and parallel worksheets after the session. The SLCT was used in a similar design in an Indian population, indicating the validity of the task, to study the immediate effects.[8]

Throughout the CM practice, the subjects kept their eyes closed and followed pre-recorded instructions. The instructions emphasized carrying out the practice slowly, with awareness and relaxation. The practice began by repeating a verse (40 seconds) from the yoga text, the *Mandukya Karika*;[9] followed by isometric contraction of the muscles of the body, ending with supine rest (1:00 minute); slowly coming up on the left side and standing at ease (called *tadasana*) and ‘balancing’ the weight on both feet, called centering (2:00 min); then the first actual posture, bending to the right (*ardhakaticakrasana*, 1 minute 20 seconds); a gap of 1 minute 10 seconds in *tadasana*, with instructions about relaxation and awareness; bending to the left (*ardhakaticakrasana*, 1 minute 20 seconds); a gap as before (1 minute 10 seconds); forward bending (*padahastasana*, 1 minute 20 seconds); another gap (1 minute 10 seconds); backward bending (*ardhacakrasana*, 1 minute 20 seconds); and slowly coming down in the supine posture with instructions to relax different parts of the body in sequence (10 minutes). The postures were practiced slowly, with awareness of all the sensations that are felt. The total duration of the practice was 22 minutes 30 seconds.[2]

During SR, or the ‘corpse posture’ the subjects lay supine with legs apart and arms away from the sides of the body and with their eyes closed. This practice lasted 22 minutes 30 seconds, so the duration was the same as for CM.

### Data analysis

Statistical analysis was done using SPSS (Version 10.0). The total number of wrong attempts and the net score data were analyzed using the repeated measures analyses of variance (RMANOVA). There was one Within Subjects Factor, that is, States with two levels (pre and post) and one Between Subjects Factor, that is, Groups with two levels (CM or SR session). Post-hoc tests with Bonferroni adjustment were used to detect significant differences between the mean values.

## RESULTS

The group mean values and standard deviation for total scores, scores for wrong cancellations, and net score in CM and SR sessions are given in Table 1.

**Table 1:** Total score, scores for wrong cancellation, and net score in an SLCT pre and post the CM and SR sessions. Values are in group mean and standard deviation

	Cyclic meditation		Supine rest	
	Pre	Post	Pre	Post
Total score for cancellation	39.07±12.21	44.84±13.24***@	38.67±12.16	42.87±13.16***
Score for wrong cancellation	0.53±1.39	0.68±1.62	0.66±1.96	0.58±1.78
Net score for cancellation	38.54±12.32	44.13±13.26***@	38.01±12.2	42.31±13.26***

*** *P*<0.001, RMANOVA with *Post-hoc* test Bonferroni adjustment, compared with respective pre score;

@ *P*<0.05, RMANOVA with Bonferroni adjustment between sessions, compared with post score of CM and SR;

Total scores differed significantly between States (F=222.92, *P*<0.001) and there was a significant interaction between session and state (F=6.79, *P*<0.01). Also, the net scores differed significantly between states (F=218.58, *P*<0.001) and there was a significant interaction between session and state (F=4.62, *P*<0.033). *Post-hoc* analyses showed that for both CM and SR, there was a significant increase in total scores (*P*<0.001 in both sessions) and net scores (*P*<0.001 in both sessions) compared to the respective pre-values. Also there was a significant change in post mean (*P*<0.024) values of CM and SR in both total and net scores

## DISCUSSION

The performance in the letter cancellation task improved immediately after the two yoga-based relaxation sessions, namely, CM and SR. However, the magnitude of change in the net scores after CM was 14.5% and after SR was 11.31%. The net score change in the CM session was significantly larger than the change in the SR session. There was no significant change in the wrong cancellation score after CM and SR.

In the present study, the change in the net score had a similar trend as in an earlier study that had an identical design.[7] However, in the earlier study, there was a 24.9% improvement in the net score after CM and 11.6% after SR. This difference of change could be due to the fact that the mediator in the previous study had an average experience of 15.3±13.3 months, while in the present study the subjects had undergone only a seven-day training program. These results revealed that the average duration of the practitioners had an influence on the outcome measures. For example, the progressive relaxation technique found slight differences in the first and second weeks, but major differences were observed in the fourth and fifth weeks in the ‘Smith Relaxation State Inventory’ before and after the session.[10]

Cancellation tasks involve sustained attention, concentration, visual scanning, and activation and inhibition of rapid responses.[11] Both the yoga-based relaxation techniques bring enhancement in the performance task. Another study, *Sahaja* Yoga Meditation can lead to additional improvement in executive functions, such as, manipulation of information in the verbal working memory, added improvement in the attention span, and visual-motor speed in patients with depression.[12]

Yoga practice has been understood to help in reducing anxiety, based on a reduction in the levels of psychophysiological arousal. In the earlier studies, both CM and SR, practiced for an equal period, found improvement in the metabolic cost,[2,3,5] autonomic function,[4] and attention measure, using P300.[6] Further study is required for an understanding of the mechanisms involved while forming the task and the effect of age and gender groups.
